# Breast tumor kinase and extracellular signal-regulated kinase 5 mediate Met receptor signaling to cell migration in breast cancer cells

**DOI:** 10.1186/bcr2622

**Published:** 2010-08-05

**Authors:** Nancy E Castro, Carol A Lange

**Affiliations:** 1 Department of Pharmacology, University of Minnesota, 321 Church Street S.E., Minneapolis, MN 55455, USA; 2 Department of Medicine (Division of Hematology, Oncology, and Transplantation), University of Minnesota, 420 Delaware Street S.E., Minneapolis, MN 55455, USA; 3 Masonic Cancer Center, University of Minnesota, 425 E. River Road, Minneapolis, MN 55455, USA

## Abstract

**Introduction:**

Breast tumor kinase (Brk/protein tyrosine kinase 6 (PTK6)) is a nonreceptor, soluble tyrosine kinase overexpressed in the majority of breast tumors. Previous work has placed Brk downstream of epidermal growth factor receptor (ErbB) activation and upstream of extracellular signal-regulated kinase 5 (ERK5) and p38 mitogen-activated protein (MAP) kinases. Herein we investigate the regulation of Brk kinase activity and cell migration in response to treatment of keratinocytes (HaCaT cells) and breast cancer cell lines (MDA-MB-231 and T47D cells) with hepatocyte growth factor (HGF) and macrophage stimulating protein (MSP), peptide ligands for Met and Ron receptors, respectively.

**Methods:**

In vitro kinase assays were performed to directly measure Brk kinase activity in response to MET and RON ligands. Transfection of Brk-targeted RNAi was used to knock down endogenous Brk or ERK5 in multiple cell lines. Kinase activities (downstream of MET signaling) were assayed by Western blotting using total and phospho-specific antibodies. Boyden chamber assays were used to measure cell migration in response to manipulation of Brk and downstream MET effectors. Rescue experiments were performed by knock down of endogenous Brk using RNAi (targeting the untranslated region (3′-UTR)) and transient transfection (re-expression) of either wild-type or kinase-inactive Brk.

**Results:**

Brk gene silencing revealed that HGF, but not MSP, induced robust Brk-dependent cell migration. Brk and ERK5 copurified in HGF-induced protein complexes, and Brk/ERK5 complexes formed independently of Brk kinase activity. ERK5 was required for breast cancer cell but not keratinocyte cell migration, which became ERK1/2-dependent upon ERK5 knockdown. Notably, rescue experiments indicated that the kinase activity of Brk was not required for HGF-induced cell migration. Further, expression of either wild-type or kinase-inactive Brk in Brk-null MDA-MB-435 cells activated ERK5 and conferred increased HGF-induced cell migration.

**Conclusions:**

These results have identified Brk and ERK5 as important downstream effectors of Met signaling to cell migration. Targeting ERK5 kinase activity or inhibiting the formation of Brk/ERK5 complexes may provide an additional means of blocking cell migration associated with breast cancer progression to metastasis.

## Introduction

Breast tumor kinase (Brk), also termed protein tyrosine kinase 6 (PTK6), was cloned from a human metastatic breast tumor and is a member of a novel family of soluble or nonreceptor PTKs with approximately 56% homology to the kinase domain of c-Src [1]. Similar to c-Src, Brk contains tandem N-terminal src homology 3 (SH3) and src homology 2 (SH2) domains and a C-terminal protein kinase domain [1]. However, Brk does not appear to be myristoylated and is present in both the cytoplasm and nucleus, but differentially localized in a cell type-specific manner. Membrane-associated Brk has recently been linked to oncogenic actions in human embryonic kidney (HEK)-293 cells [2]. Brk is undetectable in normal mammary gland. However, it is overexpressed in a majority of human breast tumors and breast cancer cell lines [3-6]. In addition to human breast tumors, elevated Brk expression has also been demonstrated in colon tumors [7], melanoma [8], lymphoma [9] and ovarian cancer cell lines [10]. Brk is present in the nuclei of normal prostate cells and well-differentiated prostate cancer cells, but mislocalized (more cytoplasmic) in undifferentiated or aggressive prostate cancer cells [11].

A small number of cytoplasmic and nuclear Brk substrates have been identified and include adaptor proteins (breast kinase substrates-1 and -2, paxillin, IRS-4, GAP-A.p65), transcription factors (STAT3/5) and RNA-interacting proteins (src-associated during mitosis (Sam68) and SLM-1/2), including nucleic acid binding protein (PSF) [12-18]. The first identified substrate for Brk phosphorylation *in vivo *was Sam68 (Src-associated during mitosis, 68 kDa) [15]. Brk-dependent phosphorylation of Sam68 on tyrosine negatively regulates its RNA-binding function [15,19].

Brk expression in normal tissues is largely confined to differentiating epithelial cells of the gastrointestinal tract and skin, as well as secretory epithelial cells of the prostate [7,11,20]. The murine ortholog of Brk, Sik (Src-like intestinal kinase), is expressed in a similar pattern. Brk appears to sensitize nontransformed cells to apoptosis [21,22]. Sik-knockout mice exhibit elongated intestinal villi associated with activated AKT and reduced apoptosis [20,23]. Brk also appears to inhibit basal AKT activity in selected nontumorigenic cell line models, such as COS-1 cells, but not in T47D breast cancer cells [24]. Previous evidence primarily derived from cancer cell models supports the involvement of Brk as a novel downstream effector of epiderman growth factor receptor (EGFR) and other ErbB family member receptor signaling networks [24,25]. Brk expression in HB4a (human mammary luminal epithelial cells) leads to increased phosphoinositide 3-kinase (PI3K) activity via epidermal growth factor (EGF)-induced ErbB3 phosphorylation and the recruitment of p85 PI3K to ErbB3 receptors [26]. In addition, EGF and heregulin-β1 activate Brk in T47D breast cancer cells [25]. Studies using T47D cells stably expressing Brk shRNA demonstrated that Brk was required for heregulin-β1-induced activation of Rac1, p38 and extracellular signal-regulated kinase 5 (ERK5) mitogen-activated protein kinases (MAPKs), and increased cyclin D1 expression [25]. Further studies linked Brk signaling to p38 MAPK activation as required steps for EGF and heregulin-β1-induced T47D breast cancer cell migration [25]. The role of ERK5 was not examined in these studies.

Aside from studies focused on erbB receptor action, regulation of Brk signaling remains largely undefined and appears to occur in a cell type-specific manner. Herein we sought to identify additional growth factor receptor-mediated signaling pathways associated with Brk regulation. Our search identified Brk as a downstream effector of Met, a transmembrane receptor tyrosine kinase which consists of 145 kDa β- and 50 kDa α-subunits. Activation of the Met receptor by its ligand, hepatocyte growth factor (HGF), has been shown to induce increased cell migration of keratinocytes and is implicated in cancer metastasis [27]. The Met oncogene is frequently overexpressed in human solid tumors. In normal tissues, it is predominately expressed in epithelial cells and endothelial cells *in vivo *and in epithelial cell lines *in vitro *[28-30]. The main physiological function of Met is to serve as a receptor for HGF [29,30].

Activation of Met receptors via HGF binding promotes tyrosine phosphorylation of its intracellular β-chain domain and the recruitment of signaling protein complexes required for the activation of downstream signaling pathways. HGF is a mesenchymal cytokine produced mainly by fibroblasts, macrophages, and smooth muscle cells [30]. HGF is involved in proliferation, angiogenesis, branching morphogenesis, and matrix invasion and possesses both mitogenic and motogenic properties [30]. Breast cancers frequently exhibit dysregulation of HGF and Met signaling, ultimately resulting in increased tumor growth and invasion [31].

In addition to its role as a motility and invasion-inducing factor for cancer cells of epithelial origin, HGF has also been shown to contribute to the migration of normal keratinocytes during wound healing. Herein we demonstrate that Brk mediates HGF-induced cell migration downstream of Met receptors in both breast cancer cells and keratinocytes. In breast cancer cells, this occurs via an ERK5-dependent pathway and is independent of Brk kinase activity. These results provide insight into a potential mode of Brk action in metastatic breast cancer cells; we conclude that Brk/ERK5 complexes are key mediators of the migratory response to HGF. Stimulation of Brk-dependent Met signaling may also aid in wound healing related to skin injury. Targeting a similar pathway in breast cancer cells, perhaps by inhibition of ERK5 kinase activity or disruption of Brk/ERK5 complexes, may provide an effective means of blocking breast cancer metastasis.

## Materials and methods

### Cell culture

T47D cells were maintained in minimum essential medium (Gibco, Carlsbad, CA) supplemented with 10 μg/ml insulin, 1× nonessential amino acids, 1× penicillin/streptomycin, and 5% fetal bovine serum (FBS). MDA-MB-231 cells were maintained in Dulbecco's modified Eagle's medium (DMEM; Gibco) supplemented with 5% FBS, 1× penicillin/streptomycin, and 10 μg/ml insulin. HaCaT and COS cells were maintained in DMEM (Gibco) supplemented with 10% FBS and 1× penicillin/streptomycin. MDA-MB-435 cells were cultured in DMEM (Gibco) supplemented with 10% FBS, 1× penicillin/streptomycin and 10 μg/ml insulin.

### Antibodies and reagents

Phosphotyrosine antibodies (4G10) were purchased from Upstate Biotechnology, Inc. (Temecula, CA) and used at 1:1000 in phosphate-buffered saline Tween (PBST). Brk antibodies (Lots B0604/D0704) were purchased from Santa Cruz Biotechnology (Santa Cruz, CA) and used at 1:1000 in 1% milk. Total antibodies to p38, AKT, Erk1/2, ERK5 and Met were purchased from Cell Signaling Technology (Danvers, MA) and used at 1:1000 in 1% milk. Phospho-antibodies to p38 (Thr180/Tyr182), phospho-Akt (ser473), phospho-ERK5 (Thr218/Tyr220), phospho-Erk1/2 (Thr202/Tyr204), and phospho-Met (1234/1235) were purchased from Cell Signaling and used at 1:1000 in 1% bovine serum albumin (BSA), except phospho-Erk1/2, phospho-Met, and phospho-ERK5 were used at 1:1000 in 1% milk. Purified heregulin-β1 was purchased from Upstate Biotechnology, Inc. EGF was used at 20 ng/ml and purchased from Sigma (St. Louis, MO); HGF was used at 50 ng/ml and purchased from Millipore (Billerica, MA); and MSP was used at 80 ng/ml and purchased from R & D Systems (Minneapolis, MN). The Mek inhibitor (U0126) was purchased from Calbiochem (Gibbstown, NJ). Wt and km-Brk were transiently transfected into COS and MDA-MB-435 cells with Fugene 6 (Roche Indianapolis, IN).

### Brk siRNA

Brk (PTK6) siGenome SMARTpool duplex and Brk (PTK6) custom duplex targeting the Brk untranslated region (3′-UTR) were purchased from Dharmacon (Lafayette, CO) and transiently transfected into T47D, MDA-MB-231 or HaCaT cells at 50-100 nM with Effectene according to the manufacturer's instructions (Qiagen, Valencia, CA). Brk rescue experiments were performed by transiently cotransfecting cells with Brk small interfering RNA (siRNA) targeting the 3′-UTR or Brk mRNA and either flag-tagged vector (pCMV), flag-tagged wt-Brk or flag-tagged km-Brk (lacking the Brk 3′-UTR). Nonsilencing siRNA and flag-tagged (empty) vector were cotransfected as a control.

### Brk kinase assay

T47D and MDA-MB-231 breast cancer cells, and HaCaT keratinocyte cells were serum starved for 24 hr and treated with 50 ng/ml HGF at various time points. Brk kinase assays were performed as described previously [25].

### Cell migration assay

Modified Boyden chamber migration assays were performed using a 10-well or 48-well chamber. Boyden chamber migration assays using a 10-well chamber were performed as described previously [25]. T47D, MDA-MB-231, MDA-MB-435 and HaCaT cells were seeded at 3 × 10^5 ^in 60 mM dishes. The following day cells were transiently transfected with 50-100 nM of Brk siRNA. Three days posttransfection, cells were trypsinized, washed two times in serum-free media and then resuspended at 1 × 10^5 ^cells/ml in serum-free media containing 10 μg/ml collagen type I human placenta (Calbiochem). Thirty microliters of serum-free medium containing 10 μg/ml collagen I with or without 50 ng/ml HGF or 80 ng/ml machrophage stimulating protein was added to the lower chamber. A polycarbonate 12 μM pore membrane (Neuroprobe, Gaithersburg, MD) was placed between the lower and upper chambers. Cells (0.05 ml) were then added to the upper chamber. Intact chambers were incubated at 37°C, 5% CO_2 _for 6 hr. At the end of the incubation period, cells remaining in the upper chamber were removed with a cell scraper. The cells that migrated through the membrane were fixed and stained with HEMA3 staining kit (Fisher Scientific, Kalamazoo, MI). The membrane was then mounted on a glass slide, and the cells were counted at ×40 magnification using a light microscope. Samples were plated in triplicate, and three fields per well were counted. The results are representative of three individual experiments.

### Coimmunoprecipitations

HaCaT, MDA-MB-231, and COS-1 cells were serum starved for 24 hr and treated with 50 ng/ml of HGF at various time points. The cells were lysed in RIPA Lite buffer (5 M NaCl, 0.5 M Na_2_HP0_4_, 0.5 M NaH_2_P0_4_, 0.5 M EDTA, 1% Triton X, 1 M NaF, 0.2 M Na_3_V0_4_, 2 mg/ml aprotinin, 1 M BGP, 0.1 M PMSF, β-Me, and protease inhibitor cocktail tablet). ERK5 protein was immunoprecipitated from 1 mg total whole cell lysate using 1 μg ERK5 antibody conjugated to protein G-agarose beads (Roche). For the conjugation step, 1 μg ERK5 antibody or normal rabbit IgG control antibody was incubated with 30 μl protein G-sepharose for 1-2 hr at 4°C and washed three times with lysis buffer. ERK5 immunoprecipitations were incubated at 4°C for 3-4 hr. Next, immunocomplexes were washed with lysis buffer four times. The samples were resuspended in 50 μl lysis buffer and 15 μl 5× Laemmli sample buffer. Samples were boiled for 5 min and then separated by SDS-PAGE and transferred to PVDF (polyvinylidene fluoride) membranes for Western blot analysis. Brk or Flag (Sigma) specific antibodies were used for Western blotting.

## Results

### Brk mediates Met receptor signaling to ERK5

Previous reports have focused primarily on Brk kinase activity downstream of erbB family member receptor signaling [25]. To further investigate other growth factor receptors that may act upstream of Brk in normal and neoplastic cell contexts, we considered the Met receptor, which has been implicated in wound healing in skin cells and in cancer cell metastasis [27]. HaCaT (human keratinocytes) and MDA-MB-231 and T47D breast cancer cells were utilized as models. These cell lines coexpress Brk and Met receptors and represent a spectrum of transformed and invasive properties; MDA-MB-231 cells are highly invasive relative to T47D cells, whereas HaCaT cells are immortalized but model "normal" or nontumorigenic skin.

**Figure 1:**
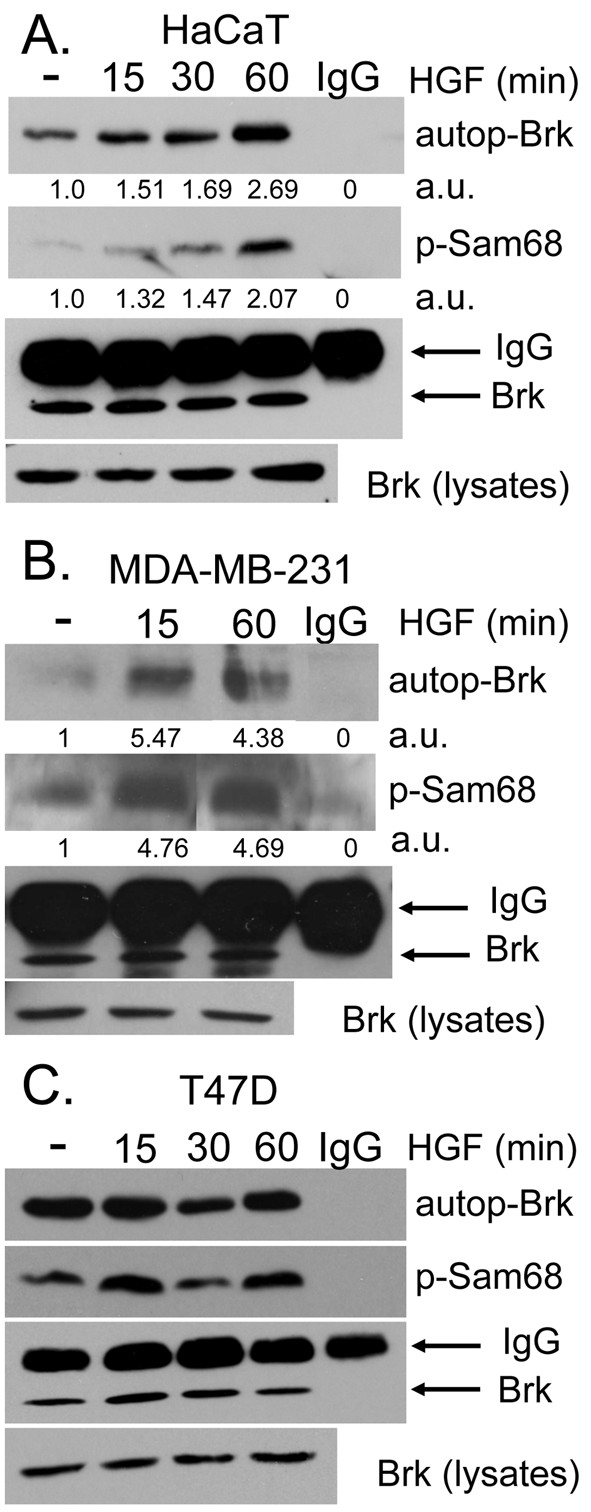
**HGF activates Brk. (a)**. *In vitro *kinase assays were performed using HaCaT cells. Serum-starved HaCaT cells were treated with either vehicle (water) or 50 ng/ml HGF for 15 to 60 min. Whole cell lysates were subjected to Brk immunoprecipitation and *in vitro *kinase assays using purified recombinant Sam68 as a substrate. Densitometry of bands represent phosphorylated Brk and Sam68 proteins indicated by arbitrary units (a.u.). Brk kinase assays were performed under similar conditions and treatments as in **(a) **using MDA-MB-231 **(b) **and T47D **(c) **breast cancer cells.

Cultures were serum starved for 24 hr and either vehicle treated or treated with HGF for 15, 30 and 60 min. Brk was immunoprecipitated from whole cell lysates using Brk-specific antibodies and subjected to *in vitro *kinase reactions containing exogenously added ATP and recombinant Sam68 as a Brk-substrate as previously defined [25]. Brk kinase activity as measured by Brk autophosphorylation and phosphorylation of its substrate, Sam68, was determined by Western blotting using a pan antiphosphotyrosine (4G10) antibody. Rabbit IgG was included as a specificity control for the IP-kinase assay. In HaCaT cells (Figure 1a), Brk exhibited weak basal activity in vehicle-treated cells relative to elevated and sustained activation throughout the HGF-induced time course. Brk autophosphorylation and *in vitro *Sam68 phosphorylation were heightened at 60 min relative to early time points (lane 4). Densitometry of bands representing phosphorylated proteins indicated a consistent time-dependent increase in Brk autophosphorylation and phosphorylation of Sam68 in HGF-treated HaCaT cells. Similar results were observed in MDA-MB-231 cells; Brk was robustly activated in response to 15-60 min of HGF exposure as measured by increased autophosphorylation of immunopurified Brk and increased phosphorylation of recombinant Sam68 *in vitro *(Figure 1b). In contrast, we observed high basal Brk activity in serum-starved T47D breast cancer cells cultured in the absence of HGF (Figure 1c). These cells appear relatively insensitive to HGF treatment; we typically observed a modest increase in Brk kinase activity, as measured by phosphorylation of Sam68 *in vitro *at 15 min of HGF treatment (Figure 1c). Equal levels of Brk were immunoprecipitated in Brk IPs; Brk was not present in IgG specificity controls. These results indicate that the Met receptor ligand, HGF, is able to activate Brk kinase activity in both keratinocytes and breast cancer cells. However, selected breast cancer cells (e.g., T47D) may contain elevated basal levels of activated Brk [25].

**Figure 2:**
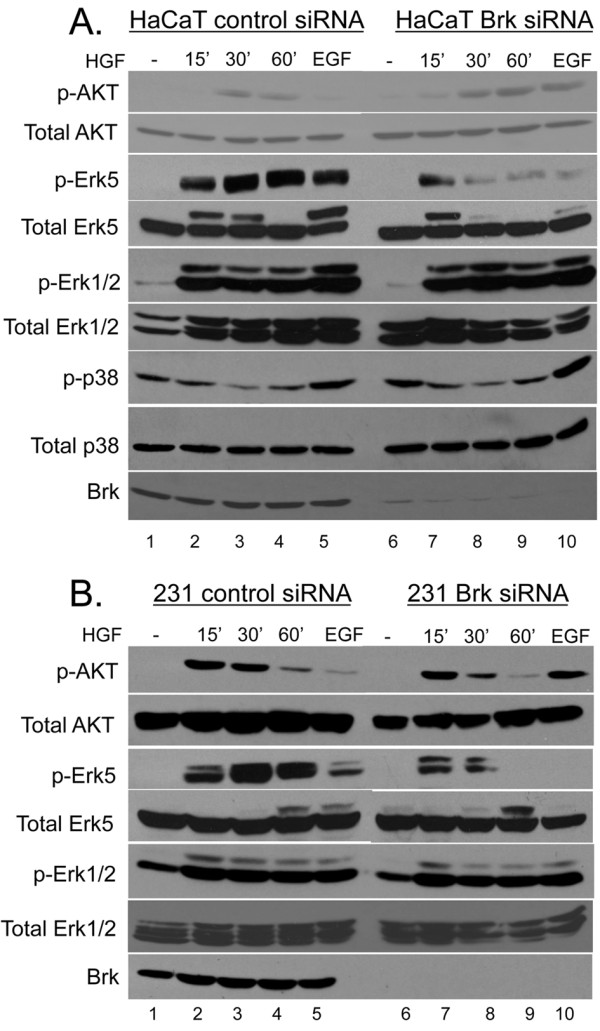
**Brk mediates Met receptor signaling to ERK5. **HaCaT **(a) **and MDA-MB-231 **(b) **cells were transiently transfected with a nontargeting siRNA (control) or Brk siRNA and serum-starved for 24 hr. Cells were then treated with vehicle (water) or 50 ng/ml HGF for 15 to 60 min. Whole cell lysates were harvested and subjected to Western blotting with phospho- and total-specific antibodies to AKT, ERK5, ERK1/2, p-38, and total Brk. Cells were stimulated with 20 ng/ml EGF for 15 min as a positive control for kinase activation.

We previously showed that Brk is required for EGF- and heregulin-induced activation of ERK5 and p38 MAPK in breast cancer cells [25]. Having established that Brk kinase activity is sensitive to HGF, we performed Brk knockdown studies to investigate whether Brk also modulates known Met receptor signaling pathways. A pool of siRNA was used to silence Brk expression in HaCaT and MDA-MB-231 cells (Figure 2). Cells were transiently transfected with either negative control or Brk siRNA. Serum-starved cells were then treated with either vehicle or HGF for 15, 30 or 60 min and whole cell lysates were analyzed by Western blotting with phospho-specific and total antibodies recognizing AKT, ERK5, ERK1/2, p38 MAPK, and Brk (Figure 2). EGF was included as a positive control for robust activation (15 min) of these kinases. HGF induced activation of AKT, ERK5 and ERK1/2 at 15-60 min in both HaCaT (Figure 2a) and MDA-MB-231 cells (Figure 2b). No significant changes in activation of p38 MAPK were observed upon HGF treatment of HaCaT cells (Figure 2a) or breast cancer cell lines (not shown); p38 MAPK was not further studied herein (except when included as a loading control). In HaCaT cells expressing Brk siRNA, phospho-AKT levels remained relatively unchanged compared to controls and throughout the HGF time course. Similarly, in MDA-MB-231 cells, Brk knockdown did not appreciably alter AKT activity in response to HGF, but increased AKT activity following EGF (Figure 2b; compare lane 5 to lane 10). These results are consistent with our previous report that Brk inhibits AKT in selected (nontransformed) cell lines [24] and studies in Sik-knockout mice, which display increased AKT activity in tissues that normally express Sik, the mouse homolog of Brk [23]. Notably, both HGF and EGF activated ERK5 at 15-60 min, as measured by either phospho-specific antibodies (recognizing Thr218 and Tyr220) or by the gel "upshift" in total ERK5. ERK5 phosphorylation, as measured using phospho-specific antibodies (Figure 2b; lanes 2-4 and 7-9) often precedes (or is separable from) gel upshift of a portion of the band representing total ERK5, which is likely due to multisite phosphorylation at sites other than (or in addition to) Thr218/Tyr220 [25,32]. Expression of Brk siRNA blocked growth factor (HGF and EGF) induced ERK5 activation (most apparent at 30-60 min) relative to control siRNA. We detected no consistent differences in the ability of these growth factors to activate ERK1/2 or in the levels of basal p38 MAPK activity in cells expressing Brk siRNA relative to control siRNA. These results suggest that Brk primarily mediates Met receptor signaling to ERK5 in breast cancer cells and keratinocytes. Brk appears to be a weak negative regulator of AKT in some cell contexts (Figure 2) [24,33].

### Brk mediates HGF-induced cell migration

**Figure 3:**
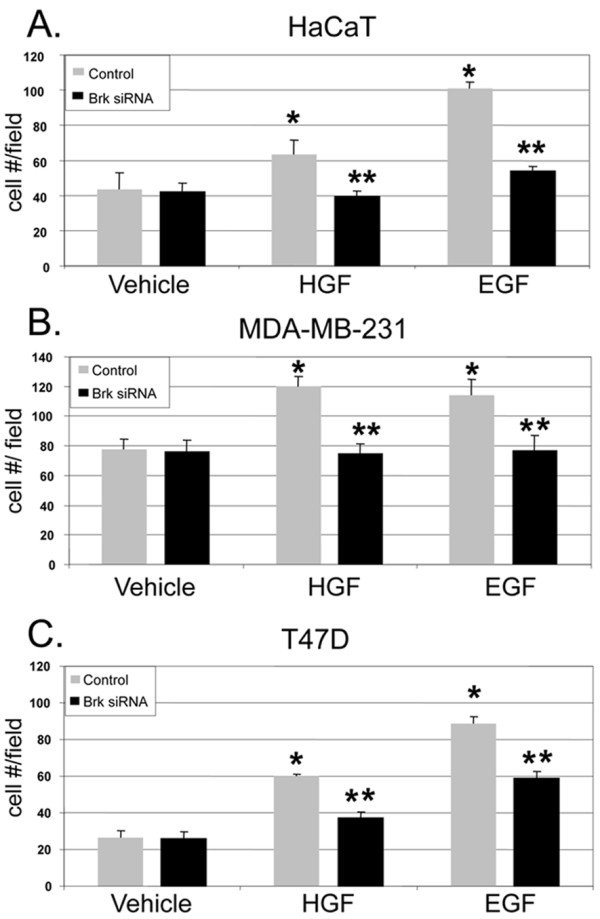
**Signaling specificity of Brk-dependent cell migration. **HaCaT **(a)**, MDA-MB-231 **(b) **and T47D **(c) **cells were transiently transfected with non-targeting (control) siRNA or Brk siRNA and either vehicle (water) or 50 ng/ml HGF was used as the chemoattractant for each Boyden chamber migration assay. As described in the Materials and Methods, migration assays were performed for 6 hr. A concentration of 20 ng/ml EGF was used as a positive control chemoattractant for migration. Error bars indicate the mean (plus standard deviation) of triplicate measures of cell migration. Single asterisks (*) denote significance (*P *< 0.05) determined by an unpaired Student's *t-*test between vehicle and HGF treated cells expressing control siRNA. Double asterisks (**) denote significance between control and Brk siRNA with either HGF or EGF used as chemoattractants. Results were confirmed in three independent experiments.

To determine whether Brk is required for HGF-induced cell migration *in vitro*, we performed Boyden chamber migration assays (Figure 3). In this assay, cells migrate from upper to lower chambers separated by cell-permeable membranes. HGF was added to the lower chamber as a chemoattractant for cells plated in the upper chamber. Cells that appear in the lower chamber over time (6 hr) are counted as a measure of induced cell migration. EGF (20 ng/ml) was used as a positive control for growth factor-induced cell migration. Both HaCaT and MDA-MB-231 cells demonstrated EGF and HGF-induced increases in migration compared to vehicle-treated control cells (Figures 3a and 3b). Similar to EGF, HGF-induced migration was attenuated in Brk siRNA-expressing cells relative to control cells, indicating a requirement for Brk in Met receptor-induced cell migration. T47D breast cancer cells were also subjected to Boyden chamber assays to demonstrate the generality of these findings (Figure 3c). Although these cells are weakly responsive to HGF-induced Brk kinase activation (Figure 1c), their HGF-induced migration is predominantly Brk-dependent.

### Signaling specificity of Brk-dependent cell migration

**Figure 4:**
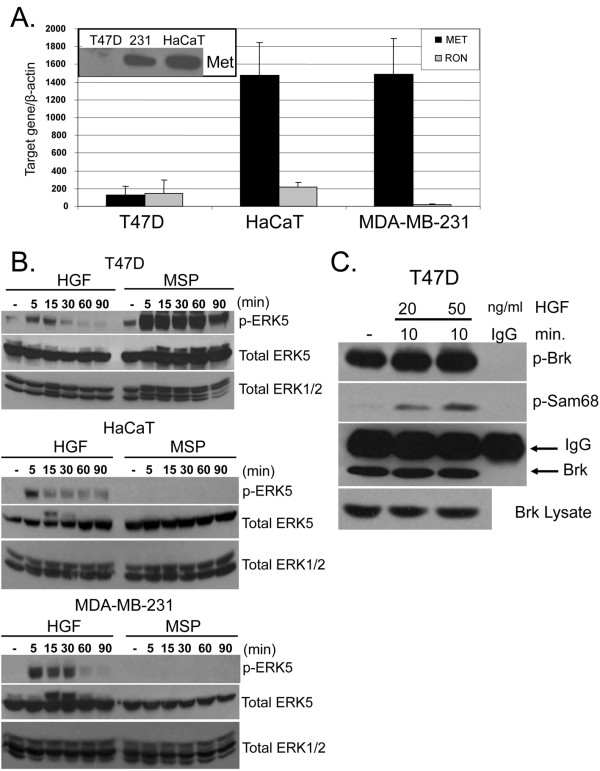
**Intact Met signaling in T47D cells. (a) **Real-time quantitative RT-PCR was performed to determine the mRNA expression levels of Met and Ron receptors in T47D, MDA-MB-231, and HaCaT cells. Error bars indicate the mean (plus standard deviation) of Met and Ron mRNA, normalized to β-actin, measured in triplicate cultures. Results were confirmed in three independent experiments. Met protein levels in all three cell lines as measured by Western blotting (insets) **(b) **T47D, HaCaT and MDA-MB-231 cells were serum-starved for 24 hr and then treated with 50 ng/ml HGF or 80 ng/ml MSP for 5 to 90 min. Western blot analyses were performed on whole cell lysates using ERK5-specific phospho- and total antibodies. ERK1/2 protein expression was used as a loading control. **(c) **T47D cells were serum-starved for 24 hr then treated with 20 or 50 ng/ml HGF for 10 min. Whole cell lysates were subjected to Brk immunoprecipitation and *in vitro *kinase assay using purified recombinant Sam68 as described in Material and Methods.

To further establish the requirement of Brk in HGF-induced cell migration we first wanted to demonstrate the specificity of our findings (Figure 3). The Met family member, Ron, is a receptor tyrosine kinase that mediates actions similar to those of the Met receptor. However, these receptors differ in ligand-binding specificity. Macrophage-stimulating protein (MSP) is a highly specific Ron ligand and does not activate Met signaling [34,35]. To measure the relative levels of Met and Ron gene expression in these cell lines, quantitative real-time PCR was performed (Figure 4a). Ron mRNA levels are similar in T47D and HaCaT cells, but greatly reduced in MDA-MB-231 cells. Notably, HaCaT and MDA-MB-231 cells have similar high levels of Met gene expression as measured by both RNA and protein (Figure 4a, inset). However, T47D cells express significantly less Met mRNA and protein in comparison to the other two cell lines, perhaps in part explaining their relative insensitivity to HGF. Although T47D cells contain ~10-fold less Met receptor relative to HaCaT and MDA-MB-231 cells, these cells are able to robustly activate ERK5 in response to HGF (5-30 min), indicating a fully functional Met signaling pathway (Figure 4b). Additionally, by increasing the sensitivity of our Brk *in vitro *kinase assay, we detected Brk kinase activity (*in vitro *phosphorylation of Sam68) following only 10 min of HGF exposure (20-50 ng/ml) while Brk autophosphorylation remained high in lysates from untreated cells (Figure 4c). In contrast to T47D cells that signal to ERK5 in response to either HGF or MSP (in the face of low mRNA expression for both receptors), both HaCaT and MDA-MB-231 cells failed to respond to MSP over a 90-min time course, while HGF remained a potent input to ERK5 activation (Figure 4b), perhaps reflective of their low levels of Ron relative to Met receptor mRNA (Figure 4a).

**Figure 5:**
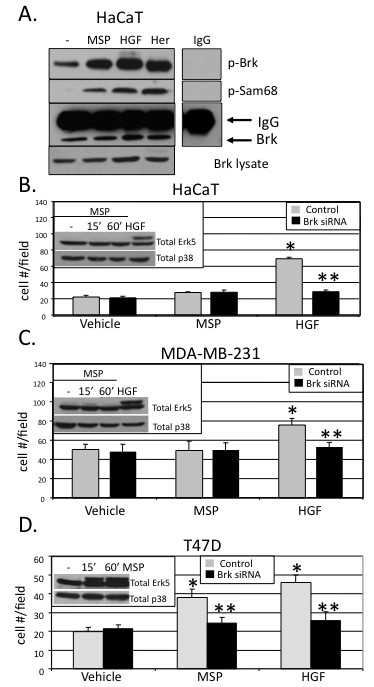
**Brk mediates cell type-specific Met and Ron receptor signaling to cell migration. (a) ***In vitro *kinase assays were performed in HaCaT cells as previously described. Cells were serum-starved for 24 hr then treated with either vehicle (water), MSP (80 ng/ml), HGF (50 ng/ml), or heregulin (25 ng/ml) for 15 min. Purified recombinant Sam68 was used as a substrate to measure Brk kinase activity. IgG control images were derived from the same experiment, Western blot, and film exposure time as the experimental lanes shown. Boyden chamber migration assays were performed in HaCaT **(b)**, MDA-MB-231 **(c)**, and T47D **(d) **cells expressing non-targeting (control) siRNA or Brk siRNA. Cells were treated with either vehicle (water) or 80 ng/ml MSP as the chemoattractant (in the lower chamber). 50 ng/ml HGF was used as a positive control chemoattractant for migration. Error bars indicate the mean (plus standard deviation) of triplicate measures of cell migration. Single asterisks (*) denote significance (*P <*0.05) determined by an unpaired Student's *t-*test between vehicle and HGF or MSP treated cells expressing control siRNA. Double asterisks (**) denote significance between control and Brk siRNA with either HGF or MSP used as chemoattractants. Results were confirmed in three independent experiments. Cells treated at various time points with 80 ng/ml MSP were subjected to Western blotting with total ERK5 specific antibody. HGF treatment was used as a positive control for ERK5 activation (insets).

Because HaCaT cells express measurable levels of Met, Ron and Her2 (our positive control), we tested the specificity of these receptors as upstream inputs to Brk activation under the same conditions by performing additional *in vitro *kinase assays (Figure 5a). When added to serum-starved HaCaT cells, all three ligands (HGF, MSP and heregulin; 15 min) activated Brk autophosphorylation and increased phosphorylation of Sam68 *in vitro*. Similar levels of Brk were immunopurified from Brk lysates (10% input) and IgG controls were clean. These data demonstrate that MSP, when added to cells that express appreciable levels of Ron receptor, can also activate Brk.

MSP expression has recently been implicated as a driver of breast cancer metastasis and is a marker of poor prognosis [36]. To test the specificity of Brk action in Ron receptor signaling to cell migration, HaCaT, MDA-MB-231 and T47D cells were transfected with either Brk siRNA or control siRNA. Boyden chamber migration assays were again performed in which cells were treated with either vehicle control, MSP or HGF (Figures 5b-d). Western blotting was performed in parallel experiments (insets to Figures 5b-d) to measure ERK5 activation (as indicated by gel upshift of a portion of total ERK5) in response to MSP (15 min). In both HaCaT and MDA-MB-231 cells (Figures 5b and 5c), MSP did not increase cell migration above basal levels. In contrast to our results with HGF, there were no differences observed with MSP-treated cells expressing control or Brk siRNA. Consistent with our above data (Figure 4b), MSP also failed to activate ERK5 relative to HGF in these two cell lines (Figures 5a and 5b, insets). In contrast, MSP both activated ERK5 (inset; and as in Figure 4b) and increased T47D cell migration above basal levels (Figure 5d). MSP-induced T47D cell migration was attenuated in Brk siRNA expressing cells. Expression of Brk siRNA again blocked HGF-induced cell migration in all three cell lines (as in Figure 3). Note that MSP can activate Brk in HaCaT cells, but it does not activate ERK5, nor do these cells migrate in response to this ligand. Taken together, these results indicate a linkage between Met/Ron signaling to Brk and the ability of a given ligand (HGF or MSP) to activate ERK5 in cell migration.

### HGF-induced cell migration requires ERK5 in breast cancer cells

**Figure 6:**
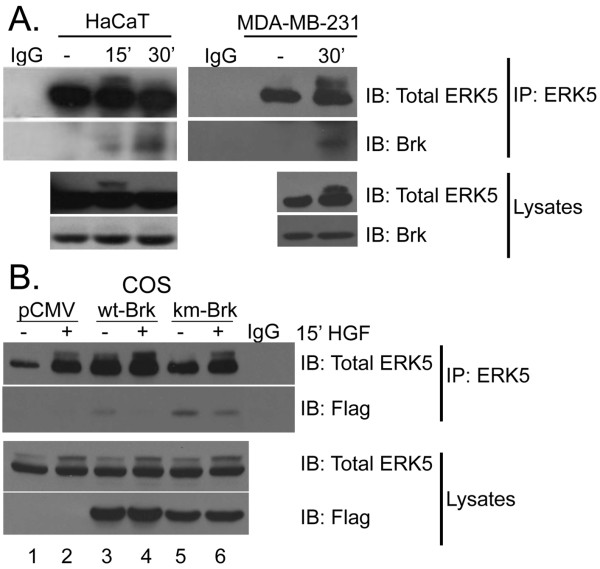
**HGF-induced cell migration requires Brk/ERK5 complexes in breast cancer cells. (a) **HaCaT and MDA-MB-231/(231) cells were treated with either vehicle or HGF for 15-30 min (30 min for 231 cells). ERK5 was immunoprecipitated from whole cell lysates using total ERK5-specific antibodies. ERK5 immunoprecipitates were then subjected to Western blotting with Brk-specific antibodies. Lysates (10% input) were Western blotted with total ERK5 and Brk-specific antibodies. **(b) **COS cells were transiently transfected with flag-tagged pCMV (vector), wt or kinase dead (km) Brk. ERK5 was immunoprecipitated from whole cell lysates using ERK5-specific antibodies and Brk was identified using flag-specific antibodies. Lysates (10% input) were Western blotted with flag antibody to indicate the efficiency of ERK5/Brk co-association.

Growth factor stimulation of breast cancer cells has been shown to alter Brk-protein complexes with signaling molecules [16,24]. To examine whether Brk and ERK5 are associated downstream of Met receptor activation by HGF, HaCaT and MDA-MB-231 cells were treated with HGF for 15-30 min (Figure 6a). ERK5 was immunoprecipitated from whole cell lysates using ERK5-specific antibodies. ERK5 immunoprecipitates were then subjected to Western blotting with Brk-specific antibodies. Endogenous Brk was present in ERK5 immunoprecipitates but was not found in IgG controls. In HaCaT cells, Brk and ERK5 interaction increased in a time-dependent manner and became readily detectable at 30 min of HGF treatment. Notably, in MDA-MB-231 cells, we also detected an HGF-regulated interaction between Brk and ERK5 following at least 30 min of HGF treatment. To test the requirement for Brk kinase activity in the Brk/ERK5 interaction, Brk-null COS-1 cells were transiently transfected with flag-tagged wt or kinase-dead (km) Brk (Figure 6b). ERK5 was again immunoprecipitated and Brk was visualized using Flag-specific antibodies. Notably, weak Brk and ERK5 association occurred following either vehicle or HGF treatment of wt- and km-Brk-expressing cells (lanes 3-6). Increased levels of km-Brk-flag were detected in ERK5 immunoprecipitates relative to wt-Brk-flag (compare lanes 5-6 to lanes 3-4). Interestingly, in COS-1 cells, decreased levels of Brk were also detected in ERK5 immunoprecipitations from HGF-treated cells (compare lanes 3-4 to lanes 5-6). These results are in contrast to the behavior of endogenous protein partners above (Figure 6a) but reminiscent of previous studies reporting the disassociation of Brk/AKT protein complexes in response to EGF treatment of HaCaT and COS-1 cells [24]. Similarly, Lukong *et al. *[16] recently reported a transition of Brk-protein complexes from large to small molecular weight upon EGF stimulation of Brk-positive BT-20 breast cancer cells. Taken together, these results suggest that Brk/ERK5 complexes are regulated by HGF in cells expressing endogenous proteins; Brk complex formation is likely cell type-dependent and may be altered by Brk overexpression.

**Figure 7:**
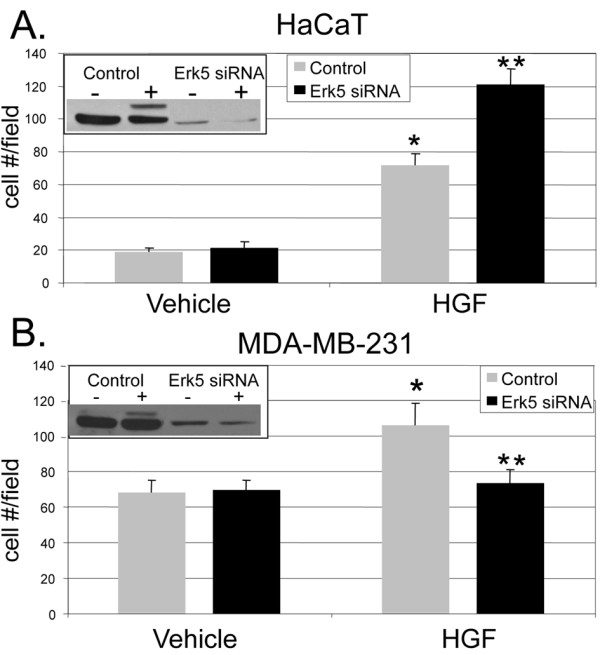
**HGF-induced cell migration requires ERK5 in breast cancer cells. **Boyden chamber migration assays were performed on HaCaT **(a) **and MDA-MB-231 **(b) **cells as previously described. Cells were transiently transfected with either non-targeting (control) siRNA or ERK5 siRNA. Lower chambers were supplemented with either vehicle (water) or 50 ng/ml HGF as the chemoattractant. Boyden chamber migration assays were performed for 6 hr. Error bars indicate the mean (plus standard deviation) of triplicate measures of cell migration. Single asterisks (*) denote significance (*P *< 0.05) determined by an unpaired Student's *t-*test between vehicle and HGF treated cells expressing control siRNA. Double asterisks (**) denote significance between control and ERK5 siRNA with HGF used as the chemoattractant. Results were confirmed in three independent experiments. Cells treated for 15 min with HGF were subjected to Western blotting; ERK5 activation and protein expression were determined using total ERK5 specific antibodies (insets).

We previously identified an important role for Brk in EGF- and heregulin- induced ERK5 activation in T47D breast cancer cells [25]. In addition, ERK5 activation has been shown by other groups to be associated with prostate cancer cell migration [37,38]. Because our studies place Brk signaling upstream of ERK5 activation in response to HGF (Figure 2), and these proteins associate upon HGF treatment (Figure 6), we wanted to further investigate the role of ERK5 signaling downstream of Met receptor activation. To test the requirement of ERK5 in HGF-induced cell migration, we again performed Boyden chamber migration assays in which cells were transiently transfected with ERK5 or negative control siRNA. Serum-starved HaCaT and MDA-MB-231 cells were treated with vehicle or HGF. Interestingly, in HaCaT cells (Figure 7a), we observed an increase in HGF-induced cell migration of cells expressing ERK5 siRNA relative to control siRNA, whereas HGF-induced MDA-MB-231 cell migration was completely blocked by ERK5 siRNA (Figure 7b). ERK5 was rapidly activated by HGF (15 min), and ERK5 expression was effectively silenced in both cell models (insets).

**Figure 8:**
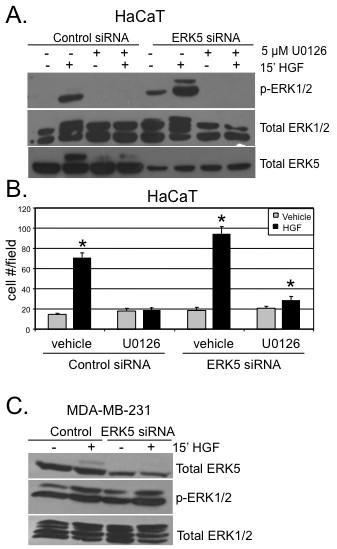
**ERK5 siRNA expressing cells signal to ERK1/2 to mediate HGF-induced cell migration in HaCaT cells. (a) **HaCaT cells expressing non-targeting (control) siRNA or ERK5 siRNA were pre-treated for 30 min with 5 μM U0126, then treated for 15 min with HGF and Western blot analysis was performed using phospho- and total-specific antibodies specific for ERK1/2 or total ERK5. **(b) **Boyden chamber migration assays were performed using HaCaT cells expressing either non-targeting (control) siRNA or ERK5 siRNA. Cells were treated with either vehicle (water) or 50 ng/ml HGF and in the presence or absence of 5 μM U0126 in the lower and upper chambers. Error bars indicate the mean (plus standard deviation) of triplicate measures of cell migration. Single asterisks (*) denote significance (*P <*0.05) determined by an unpaired Student's *t *test between vehicle and HGF treated conditions in either control or ERK5 siRNA expressing cells. Results were confirmed in three independent experiments. **(c) **MDA-MB-231 cells were transiently transfected with control siRNA or ERK5 siRNA. Cells were serum starved for 24 hr and then treated with vehicle or HGF at 15 min. Whole cell lysates were subjected to Western blotting with total-ERK5, phospho-ERK1/2 or total-ERK1/2 antibodies. ERK1/2 protein expression served as a loading control.

We were surprised to observe increased HGF-induced HaCaT cell migration upon ERK5 knockdown. However, MAPK pathways are known to be quite flexible, and there is the potential for extensive cross-talk between MAPK modules [39]. To investigate whether decreased expression of ERK5 induced compensatory activation of other closely related MAPKs, we measured total and phospho-ERK1/2 in HaCaT cells following expression of either control or ERK5 siRNA and short-term treatment with HGF (Figure 8a). As we suspected, HGF (15 min) induced more robust activation of ERK1/2 in HaCaT cells expressing ERK5 siRNA relative to cells expressing control siRNA. ERK2 (p42) was weakly active in the complete absence of growth factor stimulation (lane 5). No changes in JNK or p38 MAPK were observed under the same conditions (not shown). The Mek inhibitor, U0126 (5 μM), was included to demonstrate efficient inhibition of ERK1/2 signaling in these conditions. Total ERK5 levels indicated effective knockdown, while total ERK1/2 served as a loading control.

We again performed Boyden chamber migration assays in HaCaT cells expressing either control siRNA or ERK5 siRNA. In this set of experiments, we included U0126 (5 μM) in the top and bottom chambers for sustained ERK1/2 inhibition. This inhibitor also targets MEK5, leading to ERK5 inhibition [40]. Control siRNA- and ERK5 siRNA-expressing cells were treated with or without HGF in the presence or absence of U0126. As previously observed, ERK5 siRNA-expressing cells treated with HGF demonstrated increased migration relative to cells expressing control siRNA (Figure 8b). Interestingly, when pretreated with U0126, cell migration was attenuated back to basal levels in cells treated with HGF and expressing either control siRNA or ERK5 siRNA. Taken together, these results demonstrate a requirement for ERK5 in HGF-induced breast cancer cell migration. Keratinocytes are capable of switching from ERK5 to the closely related MAPKs, ERK1/2, when ERK5 protein levels are greatly reduced; this alternate pathway is apparently not employed by ERK5-deficient MDA-MB-231 cells. Indeed, we observed no changes in HGF-induced ERK1/2 activation upon ERK5 knockdown in MDA-MB-231 cells (Figure 8c).

### Brk kinase activity is not required for HGF-induced cell migration

**Figure 9:**
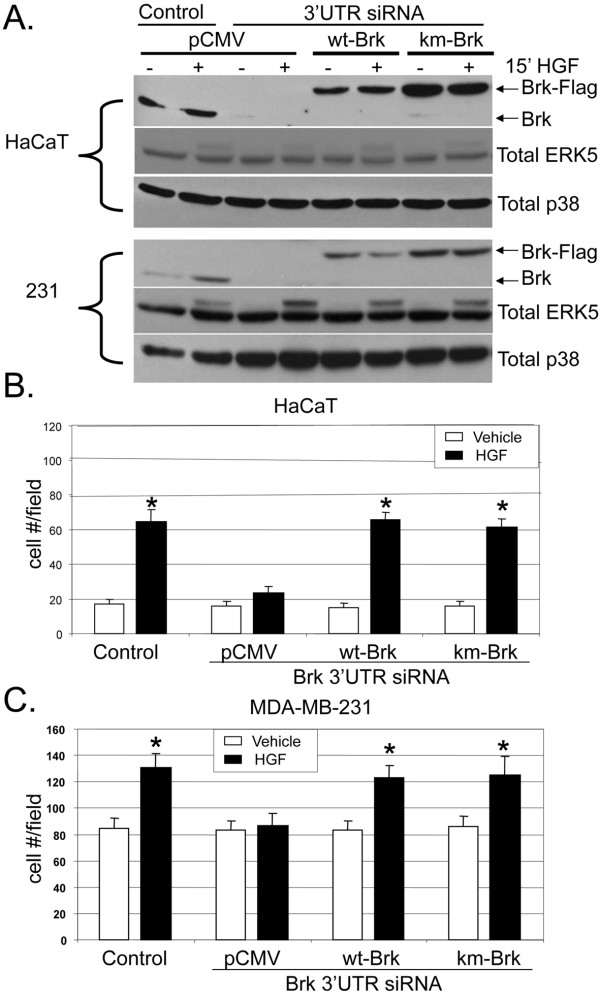
**Brk kinase activity is not required for HGF-induced cell migration. (a) **HaCaT and MDA-MB-231 (231) cells were transiently cotransfected with non-targeting (control) siRNA or Brk siRNA targeting the 3′-UTR of Brk mRNA and flag-tagged pCMV (vector), wt-Brk, or km-Brk. Western blot analysis was performed using total ERK5 and Brk-specific antibodies to identify endogenous Brk and flag-tagged Brk. p38 MAPK protein was used as a loading control. Boyden chamber migration assays were performed in HaCaT **(b) **and MDA-MB-231 **(c) **cells transiently coexpressing nontargeting siRNA or Brk 3′-UTR siRNA and either flag-tagged pCMV, wt-Brk, or km-Brk. Vehicle (water) or 50 ng/ml HGF were used as chemoattractants in the lower chamber. Error bars indicate the mean (plus standard deviation) of triplicate measures of cell migration. Single asterisks (*) denote significance (*P <*0.05) determined by an unpaired Student's *t-*test between vehicle and HGF treated conditions in either control or Brk 3′-UTR siRNA cells coexpressing wt- or km-Brk. Results were confirmed in three independent experiments.

Previous work demonstrated the ability of kinase inactive Brk to promote breast cancer cell proliferation, indicating that Brk functions in part by acting as a scaffold or adaptor molecule for active signaling complexes [41]. To test the requirement for Brk kinase activity relative to its domain structure downstream of Met receptor activation and cell migration, we performed experiments to rescue Brk knockdown. In these experiments, HaCaT and MDA-MB-231 cells were transiently transfected with either control or Brk siRNA targeting the 3′-UTR of Brk mRNA. Targeting the Brk 3′-UTR was effective in silencing native Brk mRNA, while not recognizing flag-tagged wt or kinase-inactive (km) mutant Brk transcripts (i.e., lacking the Brk 3′-UTR) that were transiently coexpressed. Thus, cells transiently expressed either flag-tagged wt-Brk or km-Brk, while silencing of endogenous Brk transcripts occurred (Figure 9a). Western blot analysis demonstrated that endogenous Brk expression levels were reduced, while wt- and km-Brk (both flag-tagged) were expressed appropriately in either HaCaT or MDA-MB-231 cells; total ERK5 levels remained similar throughout these manipulations (Figure 9a). Boyden chamber migration assays were then performed as previously described using HGF as the chemoattractant for HaCaT and MDA-MB-231 cells (Figures 9b and 9c). Consistent with the above results for Brk siRNA pools targeting the Brk open reading frame (Figure 3), and in contrast to cells expressing control siRNA, HaCaT cells expressing Brk 3′-UTR siRNA failed to migrate in response to HGF. Interestingly, HGF-induced cell migration was fully rescued in cells gene-silenced for endogenous Brk, and reexpressing either wt-Brk or km-Brk (Figures 9b and 9c). Remarkably, although cell migration was still regulated by HGF, these results demonstrate that Brk kinase activity is not required for this process, but rather Brk domain structure or scaffolding actions appear to be important (Figures 9b and 9c).

**Figure 10:**
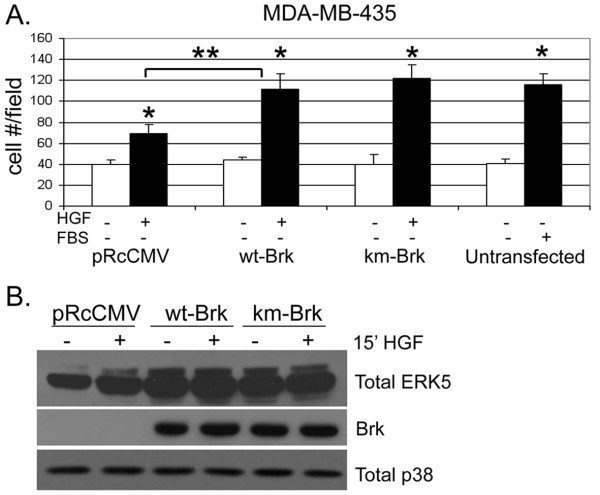
**Brk expression enhances MDA-MB-435 cell migration. (a) **MDA-MB-435 cells were transiently transfected with pRcCMV (vector), wt-, or km-Brk. Boyden chamber migration assays were performed with cells using either vehicle (water) or 50 ng/ml HGF as the chemoattractant. A concentration of 20% FBS was included as a positive control for robust migration. Error bars indicate the mean (plus standard deviation) of triplicate measures of cell migration. Single asterisks (*) denote significance (*P <*0.05) determined by an unpaired Student's *t-*test between vehicle and HGF treated conditions. Double asterisks (**) denote significance between cells expressing vector and wt-Brk with HGF as the chemoattractant. 20% FBS was used as a positive control chemoattractant. **(b) **MDA-MB-435 cells were transiently transfected with PRcCMV (vector), wt-, or km-Brk. Cells were treated for 15 min with vehicle (water) or 50 ng/ml HGF. Whole cell lysates were subjected to Western blotting with total-ERK5, Brk, and total-p38 antibodies. Brk-specific antibodies were used to demonstrate equal transfection efficiency and total endogenous p38 MAPK served as a loading control.

To further confirm that HGF-induced cell migration occurs independently of Brk kinase activity, we tested the migratory status of MDA-MB-435 cells, a Brk-null, Met-positive breast cancer cell line with similarity to melanoma [42]. MDA-MB-435 cells were transiently transfected with either wt- or km-Brk and treated with HGF in 6-hr migration assays as above. These cells were weakly migratory in response to HGF alone (Figure 10a). FBS was included as a positive control for robust migration. Similar to our results with Brk rescue (Figure 9), HGF-induced MDA-MB-435 cell migration increased upon expression of either wt or km-Brk. In both conditions, Brk expression appeared to constitutively activate ERK5 relative to vector controls as indicated by gel upshift (Figure 10b). The MEK inhibitor, U0126, when added at concentrations intended to inhibit ERK5 (10 μM), blocked wt and km-Brk-induced cell migration (not shown). These results indicate that the kinase activity of Brk is not required for Met receptor induced cell migration. Instead, our data suggest that Brk domain structure acts to recruit activated ERK5, and that both proteins are key determinants of HGF-induced cell migration.

## Discussion

Recent reports have shown Brk activation and the existence of Brk-dependent pathways in response to EGF, heregulin, and calcium signaling [25,43,44]. Brk associated with insulin-receptor substrate-4, and IGF-1 stimulated Brk phosphorylation in HEK-293 cells [18]. However, IGF-1 failed to increase the level of Brk phosphorylation or activate Brk kinase activity in T47D breast cancer cells [25]; IGF-1 effects may have gone undetected due to high basal Brk activity in these cells (Fig. 1C). Herein we sought to investigate other Brk-dependent growth factor-induced pathways crucial for processes related to breast cancer progression. We identified HGF and MSP, ligands for Met and Ron receptors, respectively, as novel ligands able to activate Brk kinase activity (Figures 1 and 5). In HGF-treated cells, we observed phosphorylation and activation of downstream kinases, including AKT, ERK1/2 and ERK5. However, following Met receptor activation, Brk acts primarily as an upstream input to ERK5 activation (Figure 2), mediating increased cell migration (Figures 3 and 7). Related to our work, Lukong and Richard [45] identified KAP3A (kinase-associated protein 3A) as a Brk substrate important for breast cancer cell migration. Notably, Brk kinase activity is not required for Brk/ERK5 interaction (Figure 6), nor HGF-induced migration upon Brk knockdown (Figure 9), or induction of migration in Brk-null cells (Figure 10). Taken together, our data suggest that Brk, acting via its domain structure or scaffolding function, may coordinate ERK5-containing signaling complexes required for HGF-induced cell migration (Figure 7). Interestingly, these complexes may contain multiple signaling molecules and can switch to ERK1/2 dependency in HaCaT cells expressing ERK5 siRNA, allowing increased migration to occur and enhancing ERK1/2 activation in the presence of HGF (Figure 8). Similar to our studies, sustained Erk1/2 activation was found to mediate epithelial cell "sheet" migration during wound healing in the Madin-Darby canine kidney (MDCK cell) model [46]. This alternate pathway is not observed in MDA-MB-231 cells upon ERK5 gene silencing (Figure 8c), perhaps revealing a vulnerability of breast cancer cells that may depend upon Brk/ERK5 complexes for their increased mobility.

Brk is a key player of Met receptor signaling upstream of ERK5, also known as BMK1 (big mitogen-activated kinase 1). Little is known about ERK5, the terminal Ser/Thr kinase of a three-kinase cascade activated by MEK5 downstream of MEKK2/MEKK3 [47]. These kinases associate via PD1-domain interactions present on both classes of kinase (MEKK and MEK). While ERK5 is believed to be exclusively activated by MEK5-dependent phosphorylation events, MEKK2/3 and MEK5 are sensitive to a variety of regulatory interactions and signaling pathway inputs [48,49]. Similar to other MAPK members, ERK5 is known to be involved in differentiation, proliferation, survival and development [50]. ERK5 can be activated by various stimuli such as stress, growth factors, mitogens, receptor tyrosine kinases and G protein-coupled receptors. Studies have associated the dysregulation of ERK5 with several human cancers; recent clinical studies have implicated elevated ERK5 in invasive breast cancer [32]. Our data demonstrate the ability of Brk and ERK5 to form a complex in HaCaT and MDA-MB-231 cells (Figure 6a). This endogenous interaction appears to be regulated in an HGF-induced manner. Brk/ERK5 complex formation occurs when ERK5 is activated in both keratinocytes and breast cancer cells; however, weak basal complex association was detected in COS-1 cells (Figure 6b). It is possible that several proteins are coassociated with Brk and Erk5 [16,24]; studies to further characterize additional components of Brk/Erk5 containing complexes are currently underway.

A correlation exists between constitutively active ERK5 and overexpression of erbB2 receptors in breast cancer cells; attenuating ERK5 expression in these cells results in inhibition of breast cancer cell growth [51]. Recent reports have identified a few ERK5 targets. These include the structural protein, connexin 43, the signaling molecule SGK (serum and glucocorticoid-inducible kinase), and transcription factors of the MEF2 (myocyte enhancer factor 2) and Sap1a (Ets-domain) families [25]. We showed previously that Brk enhanced MEF2-induced T47D cell migration in a p38 MAPK-dependent manner [25]. Our current results show that Brk also enhances HGF-induced ERK5 activation (Figures 2 and 10), implicating both kinases (i.e., Brk and ERK5) downstream of Met receptor signaling in cell migration/invasion, and suggestive of breast cancer biology related to metastasis.

The data presented here demonstrate that HGF can induce increased Brk kinase activity (Figure 1). ERK5 activation also clearly required the presence of Brk protein for signal transduction (Figure 3). As there are no pharmacologic inhibitors of Brk kinase activity, we performed knockdown and rescue experiments (Figure 9), or simply expressed Brk in Brk-null breast cancer cells (Figure 10). Notably, both wt and km-Brk rescued HGF-induced cell migration (Figure 9) and ERK5 signaling (Figure 10). Rescued (i.e., Brk expressing) cells also required HGF for cell migration. That is, Brk expression did not induce increased cell migration in the absence of HGF (Figure 9), suggesting that growth factor is required for protein complex coordination (Figure 6a). Interestingly, Brk/ERK5 complex formation does not appear to require Brk kinase activity as measured in COS-1 cells (Figure 6b) and km-Brk expression was sufficient to activate ERK5 in MDA-MB-435 cells (Figure 10b), suggesting that Brk may act primarily to scaffold the MEKK2-3/MEK5/ERK5 complex. Thus, it is important to assign functions to Brk kinase activity relative to its domain structure. Our results reveal an essential function for Brk structure, but not protein kinase activity, in HGF-induced cell migration. The underlying mechanisms as to how Brk behaves as a scaffold protein or what proteins, other than Brk and ERK5, participate in this complex remain unknown. For example, Brk may utilize its SH3 and/or SH2 domains, which have been shown to function in substrate recognition [52]; Brk structural requirements necessary for these protein interactions are likely to be complex, and remain the topic of a separate study.

In sum, Met overexpression has been linked with poor clinical outcome and implicated in breast cancer progression. Specifically, Met signaling pathways have been shown to increase tumor vasculature and volume, promote invasion of tumor cells, and increase tumor growth and survival in mouse models. Thus, it is critical to identify and target key players downstream of the Met pathway. These studies prompt further investigation of Brk and ERK5 as potential targets for the development of better treatment strategies for advanced invasive breast cancer.

## Conclusions

We have identified HGF and MSP, ligands for Met and Ron receptors, respectively, as novel regulators of Brk kinase activity. Following Met receptor activation, Brk acts primarily as an upstream input to ERK5 activation, mediating increased cell migration. Notably, Brk kinase activity is not required for Brk/ERK5 interaction or HGF-induced cell migration. We propose that Brk, acting via its domain structure or scaffolding function, coordinates activated ERK5-containing signaling complexes required for HGF-induced cell migration. Drugs that target Brk complex formation or ERK5 kinase activity may provide effective additions to breast cancer treatment regimens aimed at blocking metastasis.

## Abbreviations

Brk: breast tumor kinase; BSA: bovine serum albumin; EGF: epidermal growth factor; EGFR (erbB): epidermal growth factor receptor; ERK1/2: extracellular-signal-regulated kinase 1/2; ERK5: extracellular-signal-regulated kinase 5; FBS: fetal bovine serum; HER2 (erbB2): human epidermal growth factor receptor 2; HER3 (erbB3): human epidermal growth factor 3; HGF: hepatocyte growth factor; IGF: insulin growth factor; km-Brk: kinase mutant-Brk; MAPK: mitogen-activated protein kinase; MEK5: MAPK/ERK kinase 5; MSP: machrophage stimulating protein; PBST: phosphate-buffered saline Tween; PI3K: phosphoinositide 3-kinase; PTK6: protein tyrosine kinase 6; Sam68: src-associated during mitosis; SH2: src homology 2 domain; SH3: src homology 3 domain; Sik: src-like intestinal kinase; 3′-UTR: untranslated region; wt-Brk: wild-type-Brk.

## Competing interests

The authors declare that they have no competing interests.

## Authors' contributions

NEC performed all the experiments. NEC and CAL contributed to experimental design, interpretation, and manuscript writing. Both authors read and approved the final manuscript.
